# Leptin attenuates D_2_ receptor‐mediated inhibition of putative ventral tegmental area dopaminergic neurons

**DOI:** 10.14814/phy2.13631

**Published:** 2018-04-02

**Authors:** Takami Murakami, Munechika Enjoji, Susumu Koyama

**Affiliations:** ^1^ Department of Clinical Pharmacology Faculty of Pharmaceutical Sciences Fukuoka University Fukuoka Japan; ^2^ Department of Advanced Pharmacology Daiichi University of Pharmacy Fukuoka Japan

**Keywords:** Brain slice, extracellular recording, receptor interaction

## Abstract

Obesity causes hyperleptinemia. We have previously shown that D_2_ receptor‐mediated inhibition of ventral tegmental area (VTA) dopaminergic neurons is attenuated in diet‐induced mice with obesity. Consequently, we hypothesized that high concentrations of serum leptin during obesity might modulate D_2_ receptor‐mediated effects on VTA dopaminergic neurons. To investigate our hypothesis, we examined leptin effects on D_2_ receptor‐mediated inhibition of putative VTA dopaminergic neurons from lean mice using electrophysiological techniques. Leptin (100 nmol/L) directly inhibited spontaneous firing in 71% of putative VTA dopaminergic neurons (leptin‐responsive), whereas the remaining 29% of neurons were leptin‐nonresponsive. In 41% of leptin‐responsive neurons, leptin attenuated the reduced firing rate produced by quinpirole (100 nmol/L), whereas the remaining 59% of neurons exhibited no effect of leptin. In leptin‐nonresponsive neurons, no significant leptin‐induced effect was observed on reduced firing rate produced by quinpirole. In leptin‐responsive neurons with positive leptin‐induced attenuation of quinpirole effects, leptin‐induced attenuation persisted for >20 min, whereas no such persistent attenuation was observed in other types of neurons. In conclusion, leptin attenuates D_2_ receptor‐mediated inhibition in a subpopulation of putative VTA dopaminergic neurons. We suggest that leptin directly decreases, and indirectly increases, excitability of VTA dopaminergic neurons. In turn, this may contribute to a change in feeding behavior through the mesolimbic dopaminergic system during the development of obesity.

## Introduction

Obesity is one of the most important public health issues in modern society. It is a risk factor for metabolic syndrome, impaired glucose tolerance, and type 2 diabetes mellitus. Furthermore, a clinical study reported association of leptin receptor polymorphisms in severely obese individuals who exhibit hyperleptinemia (Carpenter et al. 2013). Leptin is a 146 amino acid protein secreted from white adipose tissue into the circulating blood. Leptin is critical for regulation of energy intake and expenditure, and contributes to food reward through the mesolimbic dopaminergic system (Hommel et al. 2006; Domingos et al. 2011). Histochemical studies have shown that functioning leptin receptors are expressed in a subpopulation of ventral tegmental area (VTA) dopaminergic neurons (Figlewicz et al. 2003; Fulton et al. 2006; Hommel et al. 2006; Scott et al. 2009; Leshan et al. 2010).

The mesolimbic dopaminergic system, which projects from the VTA to the nucleus accumbens (NAcb), amygdala, hippocampus, and other brain regions, contributes to the reinforcement of food stimuli (Wise 2002; Robinson and Berridge 2003; Fulton 2010; Narayanan et al. 2010). Dopamine is a key neurotransmitter involved in the hedonic aspect of feeding, but is not necessarily associated with maintenance of the balance between energy intake and consumption. Animal studies have suggested that the interaction between D_2_ and leptin receptors is important for feeding regulation (Davis et al. 2009; Kim et al. 2010; Billes et al. 2012). For example, leptin reduced D_2_ receptor‐dependent food consumption in mice (Billes et al. 2012). Moreover, D_2_ receptor stimulation of the mesolimbic dopaminergic system reduced overeating in leptin receptor‐deficient Zucker rats (Davis et al. 2009). We have previously reported that D_2_ receptor‐mediated inhibition of VTA dopaminergic neurons is attenuated in high‐fat diet‐induced obese mice (Koyama et al. 2014). Taken together with the previous clinical and animal studies, we hypothesized that high concentrations of serum leptin during obesity might modulate D_2_ receptor‐mediated effects on VTA dopaminergic neurons.

Previous electrophysiological studies using whole‐cell configurations have reported that leptin reduces spontaneous firing of VTA dopaminergic neurons (Hommel et al. 2006). Furthermore, this neuronal response was triggered by janus kinase‐2–extracellular signal‐regulated kinase (JAK‐2–ERK) intracellular signal transduction (Trinko et al. 2011). Because several protein phosphorylation pathways convey intracellular signals after leptin receptor activation, it is important that physiological intracellular conditions of VTA dopaminergic neurons are maintained when examining leptin‐induced effects on electrophysiological properties of these neurons. Crucially, no study has been published to date that satisfies this experimental requirement. Thus, in this study, we investigated leptin effects on D_2_ receptor‐mediated inhibition of VTA dopaminergic neuronal excitability using an extracellular recording technique that does not disrupt the intracellular milieu (Koyama et al. 2013, 2014).

## Materials and Methods

### Animals

Male 4‐week‐old imprinting control region (ICR) mice (Kyudo Co. Ltd., Saga, Japan) were housed in groups of four in plastic cages (30 × 25 × 18 cm) under a 12/12 h light–dark cycle (lights on at 19:00). Mice were kept in a temperature‐ and humidity‐controlled room (20–24°C and 53–57%, respectively) under specific pathogen‐free conditions and with food and water available *ad libitum*. Mice were fed a standard diet (13% fat, 60% carbohydrate, and 27% protein, total energy, 3.5 kcal/g; CE‐2; Clea Japan Inc., Tokyo, Japan) for 5–6 weeks. Animals used in this study were treated in strict accordance with the U.S. National Institutes of Health Guide for the Care and Use of Laboratory Animals, and all experimental methods were approved by the Animal Care Committee of Fukuoka University.

### Preparation of brain slices

Under pentobarbital anesthesia (50 mg/kg), each mouse (9–10 weeks old) was decapitated and the brain quickly removed. The brain was placed in an ice‐cold cutting solution consisting of (in mmol/L): 220 sucrose, 2.5 KCl, 2.4 CaCl_2_, 1.3 MgSO_4_, 1.24 NaH_2_PO_4_, 26 NaHCO_3_, 11 D‐glucose, and 0.4 ascorbic acid, which was constantly bubbled with 95% O_2_ and 5% CO_2_. Transverse brain slices (400 *μ*m thick) were cut using a vibrating blade tissue slicer (7000 SMZ; Campden Instruments, Loughborough, UK). Brain slices were incubated in artificial cerebrospinal fluid (ACSF), consisting of (in mM): 126 NaCl, 2.5 KCl, 2.4 CaCl_2_, 1.3 MgSO_4_, 1.24 NaH_2_PO_4_, 26 NaHCO_3_, and 11 D‐glucose, bubbled with 95% O_2_ and 5% CO_2_ at room temperature (20–25°C) for at least 1 h. Slices were then placed on a glass platform in a recording chamber (RC‐22C; Warner Instruments, Hamden, CT, USA) and perfused with ACSF, which was constantly bubbled with 95% O_2_ and 5% CO_2_ and warmed to 35°C using an in‐line solution heater connected to a thermostatic temperature circulator (NTT‐2200; Tokyo Rikakikai Co. Ltd., Tokyo, Japan). Temperature of ACSF in the recording chamber was monitored directly using a digital thermometer (7001H; Netsuken, Tokyo, Japan). A S‐shaped platinum frame was used to hold the brain slice in the recording chamber. The VTA (located between the interfascicular nucleus and medial lemniscus in the horizontal axis, and between the paranigral nucleus and red nucleus in the sagittal axis) was identified (Franklin and Paxinos 2008) under a binocular dissection microscope (M50; Leica, Solms, Germany).

### Electrophysiology

After approximately 1 h of perfusion with ACSF, extracellular recordings of brain slice preparations were performed, with the experimenter blinded to treatment. Extracellular voltage‐clamp recordings at a holding potential of 0 mV were used, because the recording technique adopted enables the capacitor aspect of the membrane patch to provide a low impedance pathway through the patch for fast events such as action potentials (APs). Consequently, when a current is generated, the current is leaked across the seal rather than the patch under the loose patch configuration (Perkins 2006). Spontaneous AP currents were recorded using a Multiclamp‐700B patch‐clamp amplifier (Molecular Devices, Sunnyvale, CA, USA). Microelectrodes were fabricated from glass capillaries (O.D.: 1.5 mm, I.D.: 0.86 mm; BF150‐86‐10; Sutter Instrument Company, Novato, CA, USA) on a P‐97 puller (Sutter Instrument Company). The tip resistance of each electrode was 3–7 MΩ when filled with 0.9% NaCl. A depolarizing rectangular voltage pulse of 10 mV was applied to the electrode and negative pressure gently applied to complete a loose patch with an average seal resistance of 49.5 ± 2.4 MΩ (*n *=* *65). Membrane currents were filtered at 2 kHz and acquired at a sampling frequency of 10 kHz. Data acquisition was performed using a Digidata 1440A interface with pClamp software version 10.2 (Molecular Devices).

### Identification of putative VTA dopaminergic neurons

Putative VTA dopaminergic neurons were identified by their electrophysiological and pharmacological properties, among other criteria previously described (Ungless et al. 2004; Ford et al. 2006; Chieng et al. 2011; Luo et al. 2011). These neurons had AP current duration > 1.2 msec and firing frequency (FF) < 5 Hz, and were inhibited by application of the D_2_ receptor agonist, quinpirole. VTA neurons that met all three criteria were used for analyses in this study. A concentration of 100 nmol/L quinpirole was used because it potently inhibits FF (Koyama et al. 2014).

### Drug application

Ascorbic acid, quinpirole, and recombinant mouse leptin were purchased from Sigma‐Aldrich (St. Louis, MO, USA).

Brain slices were continuously perfused with ACSF, and drugs were dissolved at final concentrations in ACSF. Drug solutions were applied by a multichannel manifold (MP‐5; Warner Instruments). Each channel was connected via tubing to a gravity‐fed reservoir. Solutions flowed constantly through one manifold channel connected to the recording chamber. Application of drug or vehicle solution was controlled by opening or closing valves connected to the reservoirs. In some experiments, low‐Ca^2+^ (0.25 mmol/L) and high‐Mg^2+^ (10 mmol/L) extracellular solution was used to minimize excitatory and inhibitory synaptic activity in putative VTA dopaminergic neurons (Honda et al. 2002).

### Data analysis and statistics

AP currents were detected from the peaks of the initial inward current components using a threshold‐searching configuration in pClamp. The duration between peaks was estimated as the interspike interval (ISI), with FF also calculated. The coefficient of variation (CV) of ISI was obtained by dividing the standard deviation of ISI by mean ISI. AP current duration was measured between initiation of the inward current component and subsequent peak outward current of the AP, as described previously (Chieng et al. 2011; Koyama et al. 2013; Malgolis et al., 2008). Recordings including AP current amplitude < 10 pA and ISI CV > 1.0 were considered unstable and not used for subsequent analyses. Recordings were also excluded from analyses when spontaneous firing did not recover after drug washout. Quinpirole and leptin were applied for 2 and 4 min, respectively. Each subsequent 1‐min‐long FF epoch was normalized against the mean of the four epochs preceding drug application. In some experiments, a 30‐s‐long or 2‐min‐long FF epoch was used for analysis. Duration of 50% FF inhibition with quinpirole was determined, producing a time course graph of each recording from putative VTA dopaminergic neurons. Duration corresponds to the time between the point at which quinpirole reduced normalized FF to 50% and that at which normalized FF recovered up to 50%.

Normality of data distribution was confirmed using the Shapiro–Wilks test. Two‐tailed Student's *t*‐test was used for comparisons between two groups. Paired *t*‐test was used to compare responses from the same neuron before and after the experimental procedure. Significant differences in quinpirole‐induced effects on normalized FF between leptin‐responsive and leptin‐nonresponsive neurons were determined by two‐way analysis of variance (ANOVA). Differences were considered statistically significant at *P *<* *0.05. Numerical values are reported as mean ± SEM. Graphing and statistical analyses were conducted using Origin8 (OriginLab, Northampton, MA, USA).

## Results

### Stability of extracellular recordings from VTA neurons

Electrophysiological parameters, FF and AP current duration, were recorded from eight neurons at 0, 12, 24, 36, and 48 min. Data from 2‐min continuous recordings were analyzed at each point. Average seal resistance of extracellular recordings was as follows: 53.2 ± 5.4, 52.8 ± 5.8, 52.7 ± 5.9, 51.8 ± 5.9, and 51.8 ± 6.1 MΩ, respectively. Average FF was as follows: 2.7 ± 0.4, 2.8 ± 0.4, 2.8 ± 0.3, 2.8 ± 0.3, and 2.8 ± 0.3 Hz, respectively. Average CV of ISI was as follows: 0.033 ± 0.005, 0.031 ± 0.007, 0.034 ± 0.005, 0.028 ± 0.005, and 0.033 ± 0.006, respectively. Average AP current duration was as follows: 1.49 ± 0.08, 1.50 ± 0.09, 1.50 ± 0.08, 1.53 ± 0.09, and 1.55 ± 0.08 msec, respectively.

### Leptin‐induced reduction of spontaneous firing

Cerebrospinal concentrations of leptin in obese human and animals are estimated to be ~0.1 nmol/L (Unger 2000; Rodrigues et al. 2002), whereas previous electrophysiological studies using brain slice preparations show clear and stable leptin effects on spontaneous firing at concentrations up to 100 nmol/L (Honda et al. 2002; Hommel et al. 2006; Trinko et al. 2011). We compared 0.1 and 100 nmol/L leptin‐induced effects on spontaneous firing of VTA neurons. Average normalized FF was reduced to 0.26 ± 0.12 by 4‐min‐long application of 0.1 nmol/L leptin (*n* = 8) and 0.03 ± 0.01 by 4‐min‐long application of 100 nmol/L leptin (*n* = 6), respectively. Normalized FF during 4‐min‐long application of 0.1 and 100 nmol/L leptin was significantly different (df = 1, *F* = 10.50, *P *<* *0.005, two‐way ANOVA). In the following experiments, we used 100 nmol/L leptin for its potent effect on spontaneous firing.

### Leptin‐induced inhibition under presynaptic blockade

**Figure 1:**
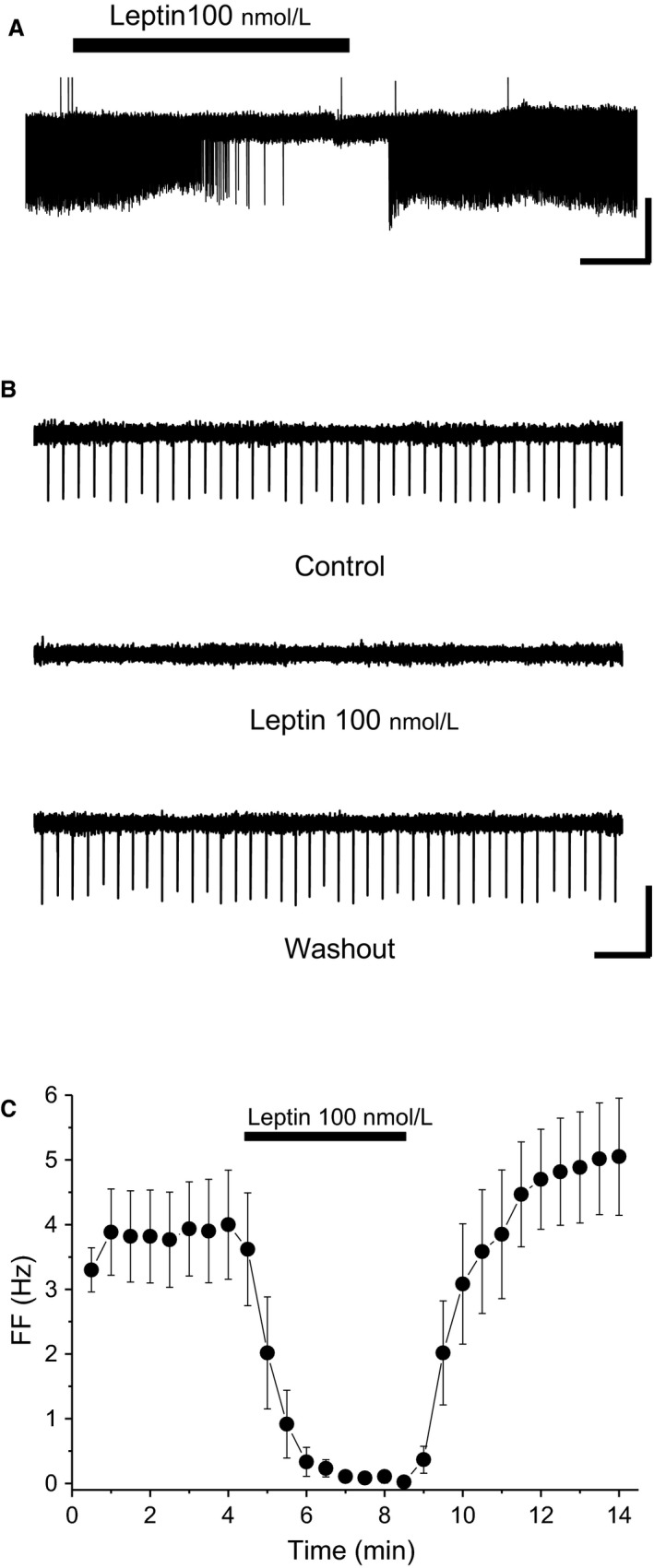
Leptin‐induced inhibition of VTA neurons. A) Spontaneous firing of ventral tegmental area (VTA) neurons before, during, and after application of 100 nmol/L leptin in low‐Ca^2+^ and high‐Mg^2+^ extracellular solution. Scale bars, 60 pA, 1 min. (B) Faster timescale of spontaneous firing of VTA neurons before, during, and after application of 100 nmol/L leptin in low‐Ca^2+^ and high‐Mg^2+^ extracellular solution. Scale bars, 60 pA, 1 sec. (C) Average time course of firing frequency (FF) before, during, and after application of 100 nM leptin in low‐Ca^2+^ and high‐Mg^2+^ extracellular solution (*n *=* *6). Each point represents FF for 30 sec. Values are mean ± SEM.

We used a low‐Ca^2+^ and high‐Mg^2+^ extracellular solution to minimize excitatory and inhibitory presynaptic transmission in VTA neurons. FF of VTA neurons was 2.9 ± 0.3 Hz in normal ACSF, and 3.8 ± 0.7 Hz in low‐Ca^2+^ and high‐Mg^2+^ extracellular solution (*n *=* *6). There was no significant difference between the two solutions (*t * = −1.34, df = 5, *P *=* *0.239). Leptin reversibly reduced FF of VTA neurons in low‐Ca^2+^ and high‐Mg^2+^ extracellular solution (*n *=* *6) (Fig. 1).

### Putative VTA dopaminergic neurons

In this study, 51 putative VTA dopaminergic neurons were analyzed: 36 leptin‐responsive neurons (71%) and 15 leptin‐nonresponsive neurons (29%). Average FF was 2.6 ± 0.2 Hz in leptin‐responsive neurons, and 2.3 ± 0.3 Hz in leptin‐nonresponsive neurons (*t *=* *0.89, df = 49, *P *=* *0.376). Average AP current duration in leptin‐responsive neurons was 1.56 ± 0.03 msec, and 1.57 ± 0.05 msec in leptin‐nonresponsive neurons (*t *=* *−0.22, df = 49, *P *=* *0.831). Table 1 shows the pharmacological properties of putative VTA dopaminergic neurons.

**Table 1:** Pharmacological properties of leptin‐responsive and leptin‐nonresponsive putative ventral tegmental area dopaminergic neurons

	Leptin Responsive	Leptin Nonresponsive	*t*	df	*P*
Neurons (*n* = 51)	36 (71%)	15 (29%)			
FF (Hz)					
quinpirole (100 nmol/L)					
1 min	1.8 ± 0.2	1.6 ± 0.3			
2 min	0.3 ± 0.1	0.6 ± 0.2	−1.26	49	0.215
leptin (100 nmol/L)					
1 min	2.1 ± 0.2	2.1 ± 0.3			
2 min	1.1 ± 0.2	2.0 ± 0.3			
3 min	0.8 ± 0.1	1.9 ± 0.3			
4 min	0.6 ± 0.1	1.9 ± 0.3	−4.69	49	<0.0001

Values are mean ± SEM. FF, firing frequency.

### Leptin‐responsive neurons

**Figure 2:**
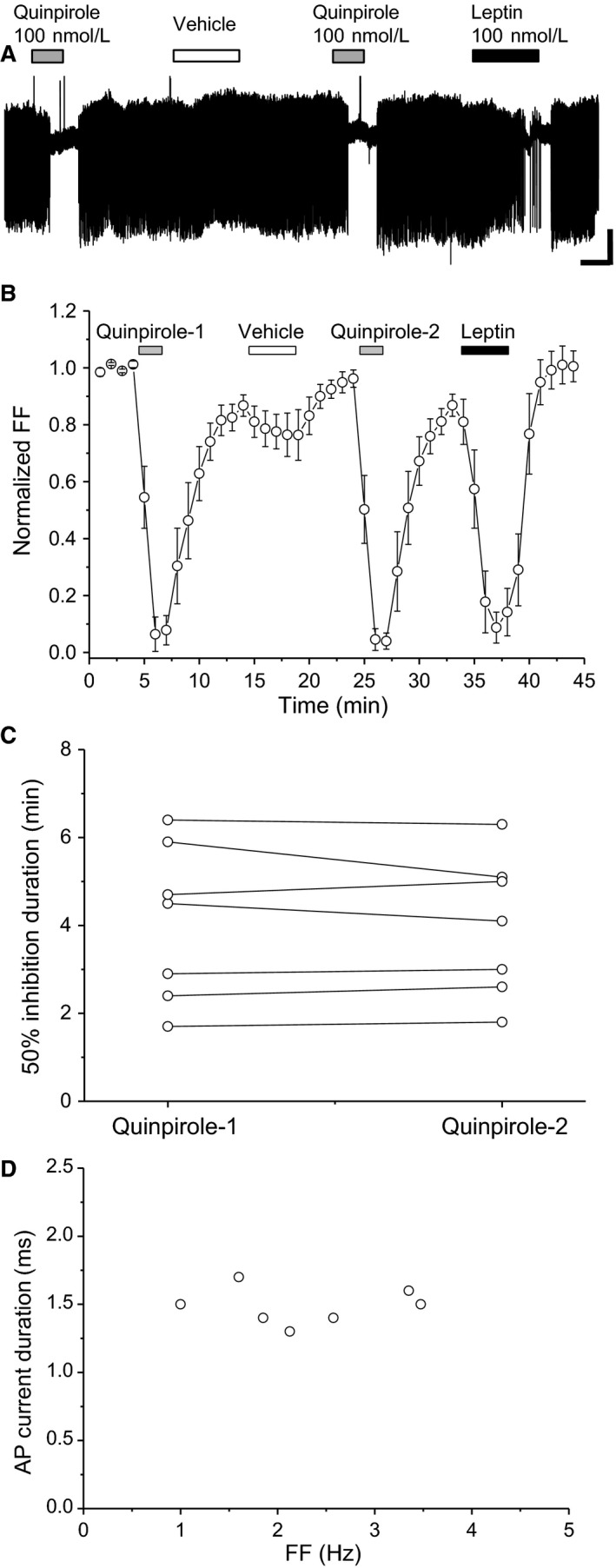
Leptin‐responsive neurons. (A) Spontaneous firing of leptin‐responsive neurons. Scale bars, 40 pA, 2 min. Quinpirole and leptin concentrations were 100 nmol/L. (B) Average time course of firing frequency (FF) (*n *=* *7). Each point represents FF for 60 sec. (C) Quinpirole‐induced first and second 50% firing inhibition durations (*n *=* *7). (D) Relationship between FF and AP current duration. Values are mean ± SEM.

Quinpirole and leptin notably inhibited FF in VTA neurons, whereas vehicle did not result in such distinct inhibition (Fig. 2A and B). In leptin‐responsive neurons, vehicle did not affect the second quinpirole‐induced effect on FF compared with the first one. Accordingly, the first and second quinpirole‐induced 50% inhibition durations were not significantly different (*t *=* *0.58, df = 6, *P *=* *0.581) (Fig. 2C). Thus, the leptin‐responsive VTA neurons examined met the criteria for putative dopaminergic neurons (Fig. 2D). From data in the vehicle experiment, we calculated the reduction rate of the second quinpirole‐induced effect against the first one. The reduction rate median value was −3.4%, with an interquartile range of 15.3%. In the following experiments, cells that exhibited reduction rates ≥20% after leptin treatment were assumed to reflect positive leptin‐induced attenuation of the second quinpirole effect. Meanwhile, cells that exhibited reduction rates <20% after leptin treatment were assumed to show negative leptin‐induced attenuation of the second quinpirole effect.

**Figure 3:**
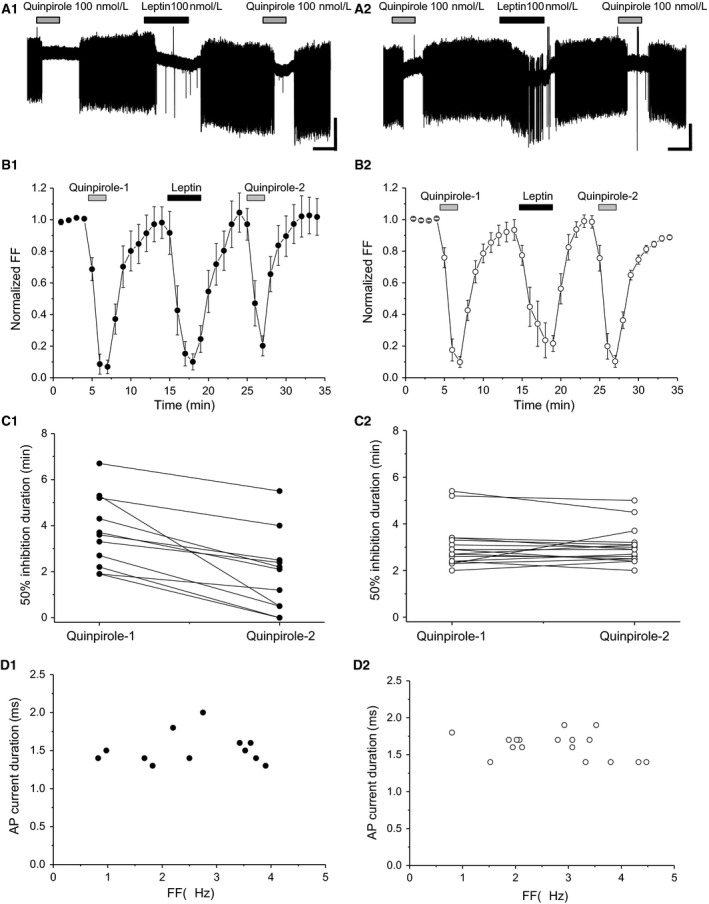
Effect of quinpirole before and after leptin exposure in leptin‐responsive neurons. (A_1_) Spontaneous firing of leptin‐responsive neurons, with positive leptin‐induced attenuation of the second quinpirole effect. Scale bars, 40 pA, 2 min. (B_1_) Average time course of firing frequency (FF) (*n *=* *12). Each point represents FF for 60 sec. (C_1_) Quinpirole‐induced 50% firing inhibition duration (*n *=* *12). (D_1_) Relationship between FF and action potential (AP) current duration (*n *=* *12). (A_2_) Spontaneous firing of leptin‐responsive neurons, with negative leptin‐induced attenuation of the second quinpirole effect. Scale bars, 40 pA, 2 min. (B_2_) Average time course of FF (*n *=* *17). Each point represents FF for 60 sec. (C_2_) Quinpirole‐induced 50% firing inhibition duration (*n *=* *17). (D_2_) Relationship between FF and AP current duration (*n *=* *17). Quinpirole and leptin concentrations were 100 nmol/L. Values are mean ± SEM.

In 12 of 29 leptin‐responsive neurons, leptin attenuated the second quinpirole‐induced effect compared with the first one (Fig. 3A_1_ and B_1_). Moreover, in these neurons, leptin significantly attenuated the second quinpirole‐induced 50% inhibition duration (*t *=* *5.31, df = 11, *P *<* *0.0001) (Fig. 3C_1_). Average normalized FF during the first and second quinpirole applications was 0.7 ± 0.1 and 1.0 ± 0.1 at 1 min, respectively, and 0.1 ± 0.1 and 0.5 ± 0.1 at 2 min, respectively (*n *=* *12). Normalized FF during the first and second 2‐min‐long quinpirole applications was significantly different in these neurons (df = 1, *F *=* *11.41, *P *<* *0.005, two‐way ANOVA). Thus, the leptin‐responsive VTA neurons examined met the criteria for putative dopaminergic neurons (Fig. 3D_1_). In 17 of 29 leptin‐responsive neurons, leptin did not attenuate the second quinpirole‐induced effect compared with the first one (Fig. 3A_2_ and B_2_). Interestingly, the time course of leptin‐induced inhibition of FF was delayed, with peak inhibition appearing at 1 min after washout of leptin (Fig. 3B_2_). In these neurons, the first and second quinpirole‐induced 50% inhibition durations were not significantly different (*t *=* *0.383, df = 16, *P *=* *0.707) (Fig. 3C_2_). Hence, the leptin‐responsive VTA neurons examined met the criteria for putative dopaminergic neurons (Fig. 3D_2_).

### Leptin‐nonresponsive neurons

**Figure 4:**
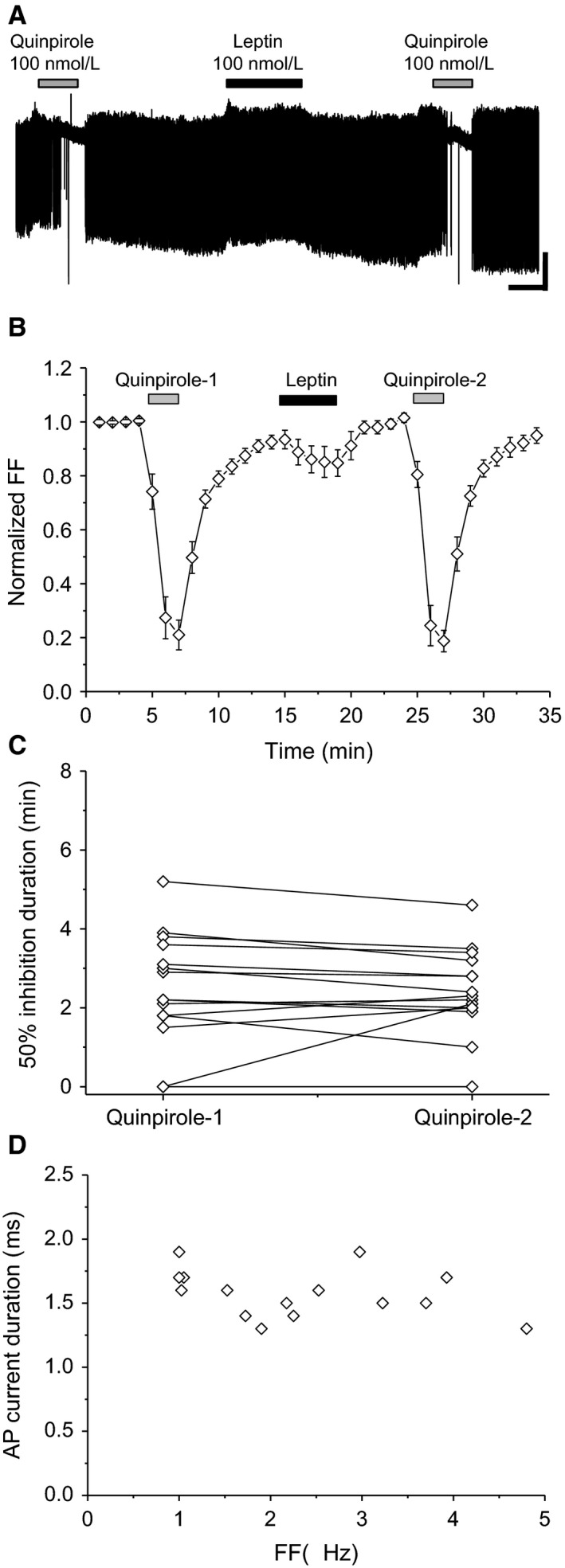
Effect of quinpirole before and after leptin exposure in leptin‐nonresponsive neurons. (A) Spontaneous firing of leptin‐nonresponsive neurons. Scale bars, 40 pA, 2 min. Quinpirole and leptin concentrations were 100 nmol/L. (B) Average time course of firing frequency (FF) (*n *=* *15). Each point represents FF for 60 sec. (C) Quinpirole‐induced 50% firing inhibition before and after leptin (*n *=* *15). (D) Relationship between FF and action potential (AP) current duration (*n *=* *15). Values are mean ± SEM.

In leptin‐nonresponsive neurons, leptin did not attenuate the second quinpirole‐induced effect compared with the first one (Fig. 4A and B). In these neurons, the first and second quinpirole‐induced 50% inhibition durations were not significantly different (*t *=* *0.327, df = 14, *P *=* *0.749) (Fig. 4C). Thus, the leptin‐nonresponsive VTA neurons examined met the criteria for putative dopaminergic neurons (Fig. 4D).

### Long‐lasting leptin‐induced suppression of D_2_ receptor‐mediated responses

**Figure 5:**
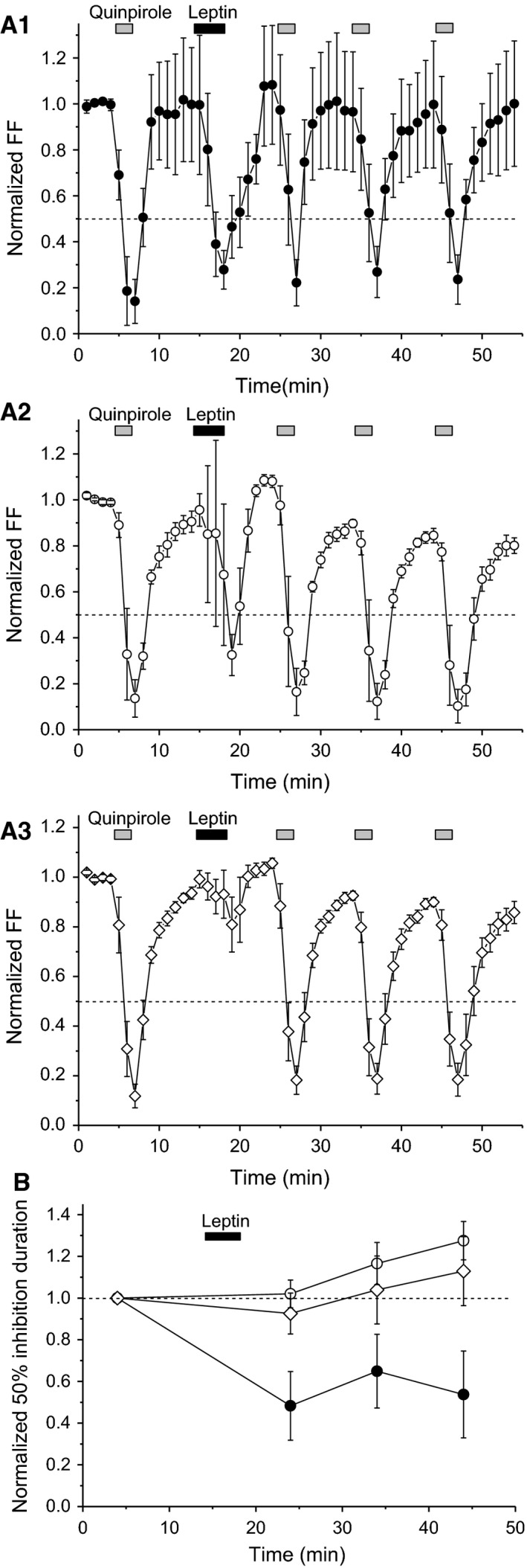
Long‐lasting leptin‐induced suppression of D_2_ receptor‐mediated responses. (A) Time course of firing frequency (FF) in leptin‐responsive neurons with positive (A_1_) or negative leptin‐induced attenuation of quinpirole effects (A_2_) and leptin‐nonresponsive neurons (A_3_). Quinpirole and leptin concentrations were 100 nmol/L. Each point represents FF for 60 sec. (B) Normalized 50% firing inhibition duration upon repeated quinpirole application in leptin‐responsive neurons with positive (closed circles) or negative leptin‐induced attenuation of quinpirole effects (open circles) and leptin‐nonresponsive neurons (open diamonds). Leptin‐responsive neurons with positive leptin‐induced attenuation of quinpirole effects (*n *=* *5). Leptin‐responsive neurons with negative leptin‐induced attenuation of quinpirole effects (*n *=* *5). Leptin‐nonresponsive neurons (*n *=* *6). Values are mean ± SEM.

Figure 5A shows the average time course of FF in leptin‐responsive and leptin‐nonresponsive neurons with repeated application of quinpirole after leptin treatment. In leptin‐responsive neurons exhibiting positive leptin‐induced attenuation of the second quinpirole effect, attenuation of the third and fourth quinpirole effects persisted (Fig. 5A_1_). In these neurons, FF after first application of quinpirole was variable, which lasted for the whole experimental duration, presumably due to wide variation in 50% inhibition durations produced by the first quinpirole effect (see Fig. 3A_1_). In contrast, leptin did not cause persistent attenuation of quinpirole effects in leptin‐responsive neurons exhibiting negative leptin‐induced attenuation of the second quinpirole effect (Fig. 5A_2_), nor in leptin‐nonresponsive neurons (Fig. 5A_3_). Leptin persistently shortened the duration of quinpirole‐induced normalized FF reduction in leptin‐responsive neurons, with positive leptin‐induced attenuation of the second quinpirole effect. Moreover, other types of neuron did not exhibit persistent leptin‐induced attenuation of quinpirole effects (df = 2, *F *=* *11.72, *P *<* *0.0001) (Fig. 5B).

## Discussion

In this study, we report two leptin receptor‐mediated effects on putative VTA dopaminergic neurons.

First, we show that leptin directly reduces spontaneous firing rate in 71% of putative VTA dopaminergic neurons. Our findings support previous immunohistochemical studies using simultaneous detection of tyrosine hydroxylase (TH) and phosphorylated signal transducer and activator of transcription 3 (STAT3) or LacZ (Figlewicz et al. 2003; Fulton et al. 2006; Scott et al. 2009). These studies show that 42–80% of VTA dopaminergic neurons express functional leptin receptors. Moreover, histochemical studies have shown that VTA dopaminergic neurons expressing leptin receptors project to the NAcb (Fulton et al. 2006) and amygdala (Leshan et al. 2010). Corroboratively, an electrophysiological study showed that quinpirole inhibited spontaneous firing of VTA dopaminergic neurons projecting to the NAcb and amygdala (Lammel et al. 2008). Therefore, leptin‐responsive neurons in this study likely correspond to VTA dopaminergic neurons projecting to these brain regions. We also found that 100 nmol/L leptin reduced FF by > 60% in putative VTA dopaminergic neurons, which was confirmed by our observation that vehicle (ACSF) failed to exhibit such clear FF inhibition in leptin‐responsive neurons. A previous electrophysiological study reported that 100 nmol/L leptin reduced the firing rate of VTA dopaminergic neurons by 20–30% in brain slice preparations (Hommel et al. 2006). This discrepancy is likely due to different recording methods: whole‐cell recordings were used in the previous study, which dilutes intracellular content, whereas we obtained extracellular recordings in our study.

Second, we show that leptin reduces the magnitude and duration of inhibition of firing rate by quinpirole in 41% of leptin‐responsive putative VTA dopaminergic neurons. Leptin‐induced attenuation of D_2_ receptor function is confirmed by the following findings from our study: (1) vehicle did not affect quinpirole‐induced responses, even in leptin‐responsive neurons; and (2) leptin did not affect quinpirole‐induced responses in leptin‐nonresponsive neurons. In our study, leptin‐induced attenuation of quinpirole‐mediated inhibition persisted for > 20 min. Since D_2_ receptor desensitization underlies long‐lasting suppression of D_2_ receptor function (Namkung and Sibley 2004; Bartlett et al. 2005), the mechanisms associated with D_2_ receptor desensitization may contribute to leptin‐induced attenuation of the effects generated by repetitive quinpirole application. In the remaining 59% of leptin‐responsive putative VTA dopaminergic neurons (which delayed the time course of leptin‐induced firing rate inhibition), leptin did not affect the magnitude nor duration of firing rate inhibition by quinpirole. Further study is needed to determine whether accelerating direct leptin‐induced reduced inhibition attenuates the effects produced by second quinpirole application.

A limitation of our study is that we did not perform neurochemical evaluation of the examined VTA neurons to determine whether they were dopaminergic neurons, for example, using TH and glutamic acid decarboxylase (GAD) as dopaminergic and GABAergic neuronal markers, respectively. We were also unable to provide information on quinpirole‐insensitive VTA dopaminergic neurons (Lammel et al. 2008; Margolis et al. 2008) or exclude VTA glutamatergic neurons, which rarely coexpress TH and GAD (Yamaguchi et al. 2007; Nair‐Roberts et al. 2008). Nevertheless, we do provide additional criteria for other studies in the literature (Ungless et al. 2004; Ford et al. 2006; Chieng et al. 2011; Luo et al. 2011), and reliably identify putative VTA dopaminergic neurons that are responsible for leptin sensitivity.

Obesity generates hyperleptinemia. We have previously reported that D_2_ receptor‐mediated inhibition of VTA dopaminergic neurons is attenuated in high‐fat diet‐induced obese mice (Koyama et al. 2014). A previous study has shown that augmentation of D_2_ receptor function in the mesolimbic dopaminergic system prevents overeating in Zucker obese rats (Davis et al. 2009). Taken together, we suggest that leptin‐induced attenuation of D_2_ receptor‐meditated inhibition of VTA dopaminergic neuronal excitability may contribute to overeating and subsequent development of obesity, and vice versa. In conclusion, leptin attenuates D_2_ receptor‐mediated inhibition in a subpopulation of putative VTA dopaminergic neurons. We suggest that leptin directly decreases, and indirectly increases, excitability of VTA dopaminergic neurons. This may contribute to a change in feeding behavior through the mesolimbic dopaminergic system during the development of obesity.

## Conflict of Interest

The authors have no competing interests to declare.

## Data Accessibility
